# Health-related quality of life among Ebola survivors in Sierra Leone: the role of socio-demographic, health-related and psycho-social factors

**DOI:** 10.1186/s12955-022-01916-y

**Published:** 2022-01-15

**Authors:** Peter Bai James, Jon Wardle, Razak M. Gyasi, Amie Steel, Jon Adams, John Alimamy Kabba, Abdulai Jawo Bah, Michael Lahai, Eugene B. Conteh

**Affiliations:** 1 National Centre for Naturopathic Medicine, Faculty of Health, Southern Cross University, Lismore, NSW 2480 Australia; 2 Faculty of Pharmaceutical Sciences, College of Medicine and Allied Health Sciences, University of Sierra Leone, Freetown, Sierra Leone; 3 African Population and Health Research Center (APHRC), Nairobi, Kenya; 4 Australian Research Centre in Complementary and Integrative Medicine, Faculty of Health, University of Technology Sydney, Ultimo, Sydney, NSW 2007 Australia; 5 Department of Pharmacy Administration and Clinical Pharmacy, School of Pharmacy, Xi’an Jiaotong University, #76 Yanta West Road, Xi’an, 710061 China; 6 Faculty of Basic Medical Sciences College of Medicine and Allied Health Sciences, University of Sierra Leone, Freetown, Sierra Leone; 7 Institute for Global Health and Development, Queen Margaret University Edinburg, Musselburgh, Scotland, UK

**Keywords:** Ebola, Ebola survivors, Health-related quality of life, SF-36, Physical heath, Mental health, Sierra Leone

## Abstract

**Background:**

Evidence of how social factors affect the health-related quality of life (HRQoL) of Ebola virus disease (EVD) survivors is limited. Our study explores the association between socio-demographic, health-related and psycho-social (stigma) factors and EVD survivors' health-related quality of life (HRQoL) in Sierra Leone.

**Methods:**

We conducted a nationwide cross-sectional study among 358 EVD survivors between January and August 2018. We used a multistage sampling method to recruit EVD survivors, and the RAND 36-Item Health Survey item was used to assess the HRQoL. Data were analysed using descriptive statistics and multiple linear regression.

**Results:**

When comparing by each dimension in relation to their respective summary scores, role limitation physical [0.00 (50.00)] and role limitation emotional [0.00 (33.33)] were the most affected physical health and mental health domains among EVD survivors respectively. EVD survivors who were older (β = − 3.90, 95% CI − 6.47 to − 1.32, *p* = 0.003), had no formal education (β = − 2.80, 95% CI − 5.16 to − 0.43, *p* = 0.021), experienced a unit increase in the number of post-Ebola symptoms (β = − 1.08, 95% CI − 1.74 to − 0.43, *p* < 0.001) and experienced a unit increase in enacted stigma (β = − 2.61, 95% CI − 4.02 to − 1.20, *p* < 0.001) were more likely to report a decreased level of physical health. EVD survivors who experienced a unit increase in the time spent in the Ebola treatment centre (β = − 0.60, 95% CI − 0.103 to − 0.18, *p* = 0.006) and those who experienced a unit increase in enacted Stigma were more likely to report decreased levels of mental health (β = − 1.50, 95% CI − 2.67 to − 0.33, *p* = 0.012).

**Conclusion:**

Sociodemographic, health-related, and psycho-social factors were significantly associated with decrease levels of HRQoL. Our findings improve our understanding of the factors that might influence the HRQoL and suggest the need for EVD survivors to be provided with a comprehensive healthcare package that caters for their physical and mental health needs.

## Background

The Ebola virus disease (EVD) is a haemorrhagic fever that is considered an emerging infectious disease with high morbidity and mortality [1]. Although recent outbreaks have occurred in East Africa [2, 3], the 2013–2016 EVD epidemic in West Africa was unprecedented, with 28,616 and 11,310 people estimated to have been infected and died respectively [4]. Despite the high morbidity and mortality of EVD, more than 10,000 patients survived EVD, and majority of the survivors suffering from physical and mental complications [5–7]. Sierra Leone was one of the most affected countries during the 2013–2016 EVD outbreak and is home to approximately 4000 survivors [8]. In addition, several Sierra Leonean studies have reported a considerable number of EVD survivors who continue to grapple with the Ebola-related physical disabilities and psychopathologies 3–4 years post-discharge from an Ebola treatment centre (ETC). Such disabilities and psychopathologies are believed to have had untoward effects on their overall quality of life [9–12].

It is important to evaluate both objective (e.g., biochemical and clinical assessment) and subjective (e.g., patient-reported health-related quality of life [HRQoL]) measures of the impact of EVD on survivors' health and well-being. HRQoL instruments evaluate a patient's own assessment of their level of functioning and satisfaction with their health and psycho-social well-being [13]. They measure individual’s physical, mental, emotional, and social functioning. Through self-appraisal, HRQoL measures also discern dysfunction and disability associated with diseases, injuries, and health behaviours at an individual and community level. HRQoL [13] has been employed to evaluate the health and psycho-social well-being of survivors of emerging and re-emerging infectious diseases [14–16]. For instance, a cross-sectional study of Middle East Respiratory Syndrome (MERS) survivors with critical illness were found to have a reduced quality of life than their counterparts with less severe illness [17]. Similarly, low HRQoL was found post-discharge among patients who had severe acute respiratory syndrome (SARS) [18] and COVID-19 [19]. As for other emerging and re-emerging infectious diseases, EVD survivors' socio-demographic, behavioural, health and psychosocial-related factors may also affect their HRQoL. For example, HRQoL was lower among MERS survivors who had a severe form of the disease [17]. Also, among COVID-19 survivors, being female and obese were associated with low mental health and physical health component of HRQOL, respectively [19]. A recent study among COVID-19 survivors in Finland found that age, sex, occupation, number of comorbidities, acute respiratory distress syndrome severity, duration of invasive mechanical ventilation were predictors of HRQoL 90 days following discharge from an intensive care unit [20].

Post-Ebola sequelae have placed limitations on survivors' ability to function in society. For instance, loss of sight, hearing and experiences of chronic pain can all lead to the inability to perform daily life activities, such as walking and running. In some cases, such physical limitations have led to survivors being unemployed, leading to dependence on others and a loss of self-worth particularly among those with advanced age [6, 21, 22]. While studies have explored the post-Ebola physical and mental health sequelae among survivors [6, 7, 9, 23], data are lacking on the impact of EVD on the overall HRQoL of survivors. Also, the current interim guideline on the management of post-Ebola sequelae among survivors is devoid of any specific reference to HRQoL due to EVD [24]. Based on studies in survivors of other emerging and re-emerging infectious disease outbreaks [17, 19, 20, 25], we hypothesised that socio-demographic, health-related and psycho-social (Ebola-related stigma) factors would influence HRQoL among EVD survivors. Therefore, this study explores the association between socio-demographic, health-related and psycho-social (stigma) factors and EVD survivors' health-related quality of life (HRQoL) in Sierra Leone.

## Methods

### Health-related quality of life theoretical framework

**Fig. 1:**
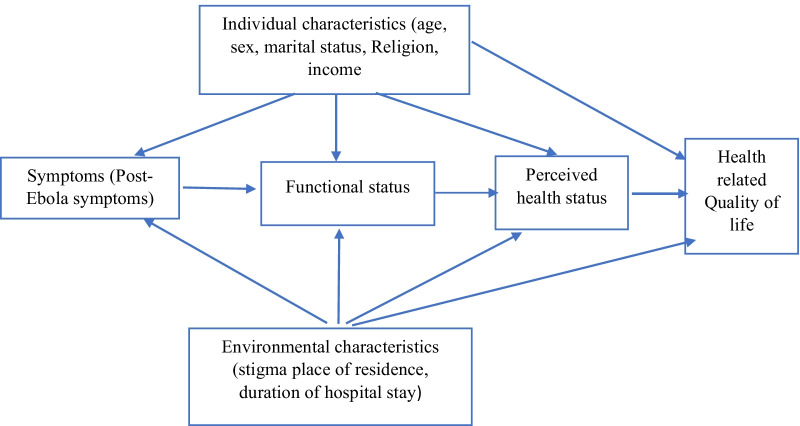
Theoretical framework underpinning how our independent variables influence Health-related Quality of life

The theoretical framework underpinning our study is based on the revised Wilson and Cleary’s HRQoL model by Ferrans et al. [26], in which they added individual and environmental characteristics to the commonly known Wilson and Cleary model [27]. We adapted the revised model by Ferrans et al. [26]. In our adapted model, socio-demographic features such as age, sex, marital status, financial status were considered to be representing the individual characteristics in the model. Health-related factors such as post-Ebola symptoms and duration of stay at the Ebola treatment centre were considered under symptoms and functional status. Also, location and stigma were considered part of environmental characteristics, whereas general health was considered under general health perceptions. The adapted model is shown in Fig. 1.

### Study design, setting, population, and sampling

**Fig. 2:**
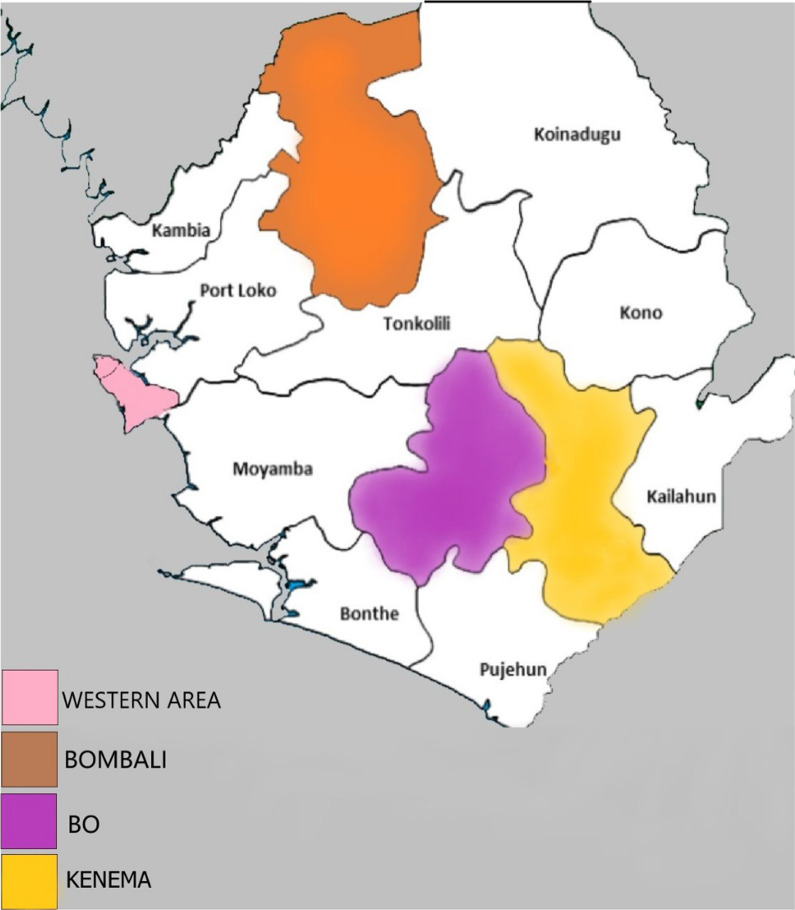
Locations of the five districts (Western area urban, Western area rural, Bombali, Bo and Kenema) in Sierra Leone. ( Source: Map created by the authors)

We conducted a nationwide cross-sectional study between January and August 2018. We conducted our study across five districts of the four administrative regions (Western Area, Northern Province, Eastern Province and Southern Province) of Sierra Leone. The locations of the five districts are shown in Fig. 2. The five districts include western area urban and western area rural districts (both in the Western area), Bo District (Southern Province), Kenema district (Eastern Province) and Bombali District (Northern Province). We chose these districts due to the high number of confirmed EVD cases reported during the outbreak, and they were host to the highest number of EVD survivors in all geographic regions in Sierra Leone [28].

We recruited adult EVD survivors 18 years or over who reported suffering from one or more post-Ebola symptoms. We excluded EVD survivors who could not provide information due to certain health and psychological limitations, such as memory loss, hearing loss, high fever and bleeding or emotional distress. A sample size formula for cross-sectional studies (N = z^2^pq/d^2^) was employed to determine the required sample size for our study, given that this study is part of an overall study that focussed on determining the prevalence of the post-Ebola symptoms among Ebola survivors. A minimum sample of 351 EVD survivors was required. However, we recruited 400 EVD survivors to allow for non-responses. We used multistage sampling to recruit EVD survivors across Sierra Leone. First, we divided the country into four geographical regions, i.e. the northern, south, east, and western regions. Secondly, we purposively chose five districts to cover all four regions. Our selection of these five districts was informed by the total number of confirmed EVD cases, and these districts had the highest number of EVD survivors. Then, we purposively chose the headquarter town or the central city as our urban area in each of the selected districts and used a lottery method to randomly choose a rural area from the list of rural settlements around the chosen urban area. In the final stage, we used simple random sampling through the lottery method to select EVD survivors. The number of EVD survivors sampled in each district was based on proportional representation using the national list of registered EVD survivors. We obtained the list from the Sierra Leone Association of Ebola survivors (SLAES). We invited 400 EVD survivors from Bombali District (Northern Region) (n = 137), Bo District (Southern Region) (n = 62), Kenema District (Eastern Region) (n = 70) and Western area urban and Western area rural districts (Western Region) (n = 131). Of those invited to participate, 377 consented to participate in the study.

### Measures

#### Demographics and health-related characteristics

We collected data on EVD survivors' age group (18–33, 34–49 and ≥ 50 years), sex (male vs. female), marital status (single, married, separated/divorce and widowed), educational status (non-formal, primary, secondary and tertiary), religious affiliation (Christianity, Islam), employment status, financial status (difficult all the time, difficult sometimes, not too bad and easy), region (north, south, east and wester), place of residence (urban and rural), known chronic condition(s) before Ebola (Yes or No), current perceived health status (very good, good, fair and poor), duration (years) since discharged from ETC, time (days) spent at ETC, number of post-Ebola symptoms, types of post-Ebola symptoms (joint pain, headache, ocular symptoms, fatigue, back pain, abdominal pain, auditory symptoms, skin disorders and alopecia). The inclusion of health-related variables was based on the available literature [7].

#### Psycho-social characteristics (Ebola-related stigma)

Ebola-related stigma was assessed using an adapted 33-item validated HIV-related stigma for people living with HIV/AIDS (HASI-P) instrument [29], given that no validated Ebola-related stigma instrument exists. The HASI-P instrument has been validated among HIV/AIDS patients in five African countries, including Lesotho, Malawi, South Africa, Swaziland, and Tanzania [29]. HASI-P consist of two major subscales, and this includes negative self-perception, which measures internalised stigma and enacted stigma. The enacted stigma subscale measures undesirable responses from the community expressed against those with the stigmatising attributes including verbal abuse, healthcare neglect, social isolation, fear of contagion and workplace stigma. We decided to use HASI-P in our study because HIV/AIDS patients and EVD survivors experience similar psycho-social difficulties regarding their social status stemming from internal and external sources [30]. There is also misinformation regarding the causes and mode of transmission of both diseases and the type of people affected in the community. We modified the HASI-P instrument to fit our local context. Following feedback from experts and a pilot test for face validity among 10 EVD survivors, we removed the two items that measured workplace stigma since close to half of EVD survivors were unemployed. In addition, given that majority of EVD survivors were not hospital inpatients after discharge from ETC, we removed one item (I was left in soiled bed") from the healthcare neglect subscale. The adapted HASI-P instrument has been published elsewhere [10]. Each of the 30 stigma items was given a score of 0–3 (0 = never, 1 = once or twice, 2 = several times and three = most of the time) to measure the frequency an EVD survivor experienced each of the investigated events. We added the scores and divided them by the number of items to get the average score of each subscale for each EVD survivor. We checked the internal consistency reliability for this instrument in this study, and the Cronbach alpha value was 0.828. Also, the Cronbach alpha values for internal and enacted stigma subscales in this study were 0.855 and 0.815 respectively.

#### Health-related quality of life

We used the RAND 36-Item Health Survey version 1.0 (SF-36v1). SF-36 is a patient self-report instrument that assessed eights domains. The domains include general health (6 items), physical functioning (10 items), role limitation due to physical health (4 items), body pain (2 items), vitality (4 items), social functioning (2 items), role limitation due to emotional problem (3 items), and mental health (4 items). RAND SF-36 instrument has been used to measure the HRQoL of survivors of emerging and re-emerging infectious diseases such as SARS, MERS, and COVID-19 [14, 17, 19]. It has also been used in African countries with good reliability measures [31–34]. We scored the items as per the instructions provided by the scoring manual [35]. First, we recoded the precoded values as per scoring instructions. Second, we calculated the mean of all items on the same domain scale to generate the eight domain scale scores. Scores for each domain ranged from 0 to 100, with the highest score representing a favourable HRQoL. The eight domains were grouped into physical (PCS) and mental (MCS) component summary scales as described by Farivar et al. [36]. PCS and MCS were calculated by multiplying the z-score of each domain scale with its respective physical and mental factor scoring coefficients (weights) and summing all eight products to obtain an aggregate physical and mental scale. These aggregate physical and mental standardised scales were then standardised by multiplying each of the aggregate by 10 and adding the resulting product to 50. Given that Sierra Leone and no other country in Africa does not have a population norm, we decided to use the United States population norm while adapting the SF-36 The scoring of the physical (PCS) and mental (MCS) component summary scales were scored using methods described by Ware et al. [37]. The internal consistency reliability for this instrument for this study was checked, and the Cronbach alpha value for the whole RAND SF-36 instrument was α = 0.918.

#### Data collection and ethical consideration

We used self-administered or interviewer-administered (for illiterate participants) formats to collect the relevant data from EVD survivors. We collected our data between May and August 2018, and it was done either in the offices of EVD survivors or homes or village courtyard. We obtained ethics approval to conduct the study from the Sierra Leone Ethics and Scientific Review Committee. EVD survivors were informed about the scope and nature of the study as well as the freedom to opt at any time. Written consent to participate in the study was obtained from each survivor before being interviewed. Consent to participate in the study was interpreted by signing or thumb printing (for illiterate participants) the consent form.

### Data analysis

We used IBM SPSS Statistics version 27 to analyse our data. We represented categorical variables using frequencies, and percentages, and median and interquartile range for continuous variables. Bivariate analysis was employed using median test (continuous variables) and Chi square test (categorical variable). We used univariate linear regression analysis to calculate the effect size (β-coefficient). Independent variables with *p* values less than 0.1 in the univariate linear regression analysis were moved into the multivariate model to determine the socio-demographic, health and psycho-social variables independently associated with, PCS and MCS. To compute the univariate and multivariate linear regression, we created dummy variables for categorical variables with more than two categories. Independent variables were considered statistically significant in the multiple linear regression analysis if their *p* values were less than 0.05.

## Results

Out of the 377 EVD survivors who consented to participate in the study, 358 completed all items of the questionnaire, and were included in the data analysis. Majority [n = 328 (91.6%)] were in the age group of 18–49 years with no significant difference between males and females [n = 128 (94.8%) vs n = 200 (89.7%), *p* = 0.115]. Close to a quarter were divorced/Separated/widowed [87 (24.3)], and more males than females were divorced/Separated/widowed [n = 87 (24.3%) vs n = 11 (8.1%), *p* = 0.001]. The median number of post-Ebola symptoms was 5.00 (3.00) and no significant difference was observed between males and females [4.00 (3.00) vs. 5.00 (3.00) *p* = 0.088]. The median time spent at the Ebola treatment centre was 21.00 (14.00) days and a gender difference was observed [males = 21.00 (15.00) vs. females = 23.00 (14.00), *p* = 0.036]. Majority reported to be experiencing joint pain [n = 319 (89.1%)] with no significant difference between males and females [n = 119 (88.1%) vs. n = 200 (89.7), *p* = 0.651]. Alopecia was reported in approximately one in ten EVD survivors [n = 38 (10.6%)] with more women [n = 30 (13.5%)] than men [n = 8 (5.9%)] reporting it. The median internalised stigma score was [median = 0.80 (IQR = 1.00)] and the median enacted stigma score was [median = 0.54 (IQR = 0.80)]. Females had higher enacted stigma than their male counterparts [median = 0.67 (IQR = 0.96) vs. median = 0.42 (IQR = 0.63); *p* = 0.005].

Table 1 provides further details.

**Table 1:** Sociodemographic, Health related, and Psycho-social (Ebola related stigma) characteristics of Ebola survivors (*N* = 358)

Characteristics	Variables	n (%) mean ± SD median (interquartile range)	Male n (%) median (interquartile range)	Female n (%) Median (interquartile range)	*p* value
*Sociodemographic characteristics*					
Age group	18–49 years	328 (91.6)	128 (94.8)	200 (89.7)	0.115
	≥ 50 years	30 (8.4)	7 (5.2)	23 (10.3)	
Religion	Christianity	92 (25.7)	27 (20.0)	65 (29.1)	0.062
	Islam	266 (74.3)	108 (80.0)	158 (70.9)	
Education status	Non-formal education	147 (41.1)	43 (31.9)	104 (46.6)	0.012
	Primary	44 (12.3)	15 (11.1)	29 (13.0)	
	Secondary	126 (35.2)	61 (45.2)	65 (29.1)	
	Tertiary	41 (11.5)	16 (11.9)	25 (11.2)	
Marital status	Single	100 (27.9)	36 (26.7)	64 (28.7)	< 0.001
	Married/cohabitating	171 (47.8)	88 (65.2)	83 (37.2)	
	Divorced/separated/widowed	87 (24.3)	11 (8.1)	76 (34.1)	
Residence	Urban	219 (61.2)	80 (59.3)	139 (62.3)	0.563
	Rural	139 (38.8)	55 (40.7)	84 (37.7)	
Economic status	Difficult all the time	110 (30.7)	42 (31.1)	68 (30.5)	0.930
	difficult some time	238 (66.5)	90 (66.7)	148 (66.4)	
	Not too bad/easy	10 (2.8)	3 (2.2)	7 (3.1)	
Region	Northern region	120 (33.5)	47 (34.8)	73 (32.7)	0.346
	Southern region	55 (15.4)	19 (14.1)	36 (16.1)	
	Eastern region	62 (17.3)	18 (13.3)	44 (19.7)	
	Western area	121 (33.8)	51 (37.8)	70 (31.4)	
Known chronic health	Yes	46 (12.8)	16 (11.9)	30 (13.5)	0.661
	No	312 (87.2)	119 (88.1)	193 (86.5)	
Employment status after surviving Ebola	Employed	199 (55.6)	66 (48.9)	133 (59.6)	0.049
	Unemployed	159 (44.4)	69 (51.1)	90 (40.4)	
*Health-related characteristics*					
Current perceived health status	Very good/good	96 (26.8)	44 (32.6)	52 (23.3)	0.055
	Fair/poor	262 (73.2)	91 (67.4)	171 (76.7)	
Time (days) spent at ETC		21.00 (14.00)	21.00 (15.00)	23.00 (14.00)	0.036
Duration (months) since discharged from ETC		42.00 (4.00)	42.00 (4.00)	42.00 (4.00)	0.922
Burden of post-Ebola symptoms		5.00 (3.00)	4.00 (3.00)	5.00 (3.00)	0.088
Arthralgia (Joint pain)	Yes	319 (89.1)	119 (88.1)	200 (89.7)	0.651
	No	39 (10.9)	16 (11.9)	23 (10.3)	
Headache	Yes	272 (76.0)	103 (76.3)	169 (75.8)	0.913
	No	86 (24.0)	32 (23.7)	54 (24.2)	
Ocular symptoms	Yes	206 (57.5)	75 (55.6)	131 (58.7)	0.554
	No	152 (42.5)	60 (44.4)	92 (41.3)	
Fatigue	Yes	181 (50.6)	73 (54.1)	108 (48.4)	0.301
	No	177 (49.4)	62 (45.9)	115 (51.6)	
Back pain	Yes	179 (50.0)	60 (44.4)	119 (53.4)	0.102
	No	179 (50.0)	75 (55.6)	104 (46.6)	
Abdominal pain	Yes	132 (36.9)	38 (28.1)	94 (42.2)	0.008
	No	226 (63.1)	97 (71.9)	129 (57.8)	
Auditory symptoms	Yes	61 (17.0)	21 (15.6)	40 (17.9)	0.561
	No	297 (83.0)	114 (84.4)	183 (82.1)	
Skin Disorder	Yes	55 (15.4)	21 (15.6)	34 (15.2)	0.937
	No	303 (84.6)	114 (84.4)	189 (84.8)	
Alopecia	Yes	38 (10.6)	8 (5.9)	30 (13.5)	0.025
	No	320 (89.4)	127 (94.1)	193 (86.5)	
*Psycho-social characteristics (Ebola-related stigma)*					
Internalized stigma (negative self-perception)		0.80 (1.00)	0.60 (1.00)	0.80 (1.00)	0.072
Enacted stigma		0.54 (0.80)	0.42 (0.63)	0.67 (0.96)	0.005

### Health-related quality of life of EVD survivors

Table 2 shows the eight domain scales of the SF-36 and their component summary scales (PCS and MCS) median scores of EVD survivors. When comparing by each dimension in relation to their respective summary scores, role limitation physical [0.00 (50.00)] and role limitation emotional [0.00 (33.33)] were the most affected physical health domain and mental health domains among EVD survivors respectively Among the eight domains, EVD survivors had the highest [52.00 (12.00)] median score for emotional wellbeing and the lowest median score for role limitation—Physical emotional [0.00 (33.33)]. Significant gender differences were observed in the physical functioning (*p* < 0,001). The mental health composite median score was [33.12 (6.79)] whilst the physical health composite median score was [30.05 (9.57)], although there was no statistically significant gender difference (*p* = 1.00).

**Table 2:** HRQOL among Ebola survivors

SF-36 domains	Median (interquartile range)	Male median (interquartile range)	Female median (interquartile range)	*p* value
General health	35.00 (20.00)	35.00 (20.00)	30.00 (15.00)	0.761
Physical Functioning	50.00 (35.00)	55.00 (30.00)	45.00 (30.00)	< 0.001
Role limitation—Physical	0.00 (50.00)	0.00 (50.00)	0.00 (50.00)	0.477
Role limitation—Emotional	0,00 (33.33)	0.00 (33.33)	0.00 (66.67)	0.129
Energy-fatigue (vitality)	45.00 (15.00)	45.00 (15.00)	40.00 (15.00)	0.094
Emotional wellbeing (mental health)	52.00 (12.00)	52.00 (12.00)	52.00 (12.00)	0.766
Social Functioning	50.00 (25.00)	50.00 (12.50)	50.00 (25.00)	0.766
Bodily pain	35.00 (32.50)	35.00 (35.00)	35.00 (22.50)	0.441
Physical Health (PCS)	30.05 (9.57)	30.70 (10.63)	29.53 (9.00)	0.275
Mental health (MCS)	33.12 (6.79)	33.18 (6.71)	33.12 (6.87)	1.000

When comparing the HRQoL by each dimension in relation to their respective summary scores, Role limitation physical was the most affected physical health domain among EVD survivors. Similarly, role limitation emotional was the was the most affected mental health domain among EVD survivors.

### Factors associated with health-related quality of life among EVD survivors

Table 3 shows details of the univariate analysis. EVD survivors who were older (β = − 6.74, 95% CI − 9.59 to − 3.89, *p* < 0.001), did not go to school (β = − 5.18, 95% CI − 7.80 to − 2.56, *p* < 0.001) widowed (β = − 3.85, 95% CI − 6.11 to − 1.60, *p* = 0.001) and have a unit increase in the number of post-Ebola symptoms (β = − 1.45, 95% CI − 1.88 to − 1.01, *p* < 0.001) were more likely to report a decreased level of PCS. Similar associations were observed for MCS. Table 4 summarises the demographic, health-related, and psycho-social factors independently associated with the physical health composite score among EVD survivors. We found that EVD survivors who were 50 years and older were more likely to report a decrease in physical health (β = − 3.90, 95% CI − 6.47 to − 1.32, *p* = 0.003), compared to those below 50 years. Also, EVD survivors with no formal education were more likely to report a decrease in physical health (β = − 2.80, 95% CI − 5.16 to − 0.43, *p* = 0.021) than participants with a tertiary education. In addition, a unit increase in the number of post-Ebola symptoms was correlated with participants’ lower physical health (β = − 1.08, 95% CI − 1.74 to − 0.43, *p* < 0.001). Further, EVD survivors who experienced a unit increase in enacted stigma were more likely to report a decreased level of physical health (β = − 2.61, 95% CI − 4.02 to − 1.20, *p* < 0.001).

**Table 3:** Univariate Analysis of the factors associated with physical health composite score, mental health composites and overall health related quality of life among Ebola survivors

Characteristics	Variables	PCS			MCS		
		β	95%CI	*p* value	β	95%CI	*p* value
Sex	Male	1.55	− 0.11, − 3.22	0.068	0.15	− 1.217, 1.509	0.833
	Female	Ref			Ref		
Age group	18–49	Ref			Ref		< 0.001
	≥ 50 years	− 6.74	− 9.59, − 3.89	< 0.001	− 4.82	− 7.15, − 2.49	
Religion	Christianity	− 0.59	− 2.45, 1.27	0.534	− 0.18	− 1.69, 1.33	0.813
	Islam	Ref			Ref		
Education status	Non-formal	− 5.18	− 7.80, − 2.56	< 0.001	− 3.70	− 5.87, − 1.54	< 0.001
	Primary	− 0.68	− 3.90, 2.53	0.676	− 0.81	− 3.47, 1.85	0.548
	Secondary	− 1.13	− 3.80, 1.53	0.404	− 1.74	− 3.94, 0.46	0.121
	Tertiary	Ref			Ref		
Marital Status	Single	Ref			Ref		
	Married/cohabitating	− 1.162	− 3.07, 0.75	0.233	− 0.13	− 1.68, 1.43	0.874
	Divorce /separated/	− 1.41	− 9.15, 6.33	0.721	2.85	− 3.45, 9.14	0.375
	widowed	− 3.85	− 6.11, − 1.60	0.001	− 2.50	− 4.33, − 0.67	0.008
Residence	urban	0.92	− 0.75, 2.58	0.281	0.72	− 0.63, 2.07	0.294
	Rural	Ref			Ref		
Economic Status	Impossible/difficult all time	− 3.73	− 8.79, 1.33	0.148	− 2.03	− 6.10, 2.05	0.329
	Difficult sometimes	− 2.19	− 7.13, 2.76		0.29	− 3.70, 4.27	
	Not too bad/easy	Ref		0.385	Ref		0.887
Region	Northern region	− 5.04	− 6.95, − 3.13	< 0.001	− 2.60	− 4.19, − 1.01	0.001
	Southern region	− 0.70	− 3.10, 1.72	0.571	− 1.18	− 3.19, 0.83	0.249
	Eastern region	− 2.49	− 4.81, − 0.18	0.035	− 1.17	− 3.10, 0.76	0.232
	Western area	Ref			Ref		
Known chronic condition	Yes	− 0.35	− 2.78, 2.08	0.777	− 0.08	− 2.06, 1.89	0.935
	No	Ref			Ref		
Employment status after surviving Ebola	Employed	Ref			Ref		
	Not employed	− 0.38	− 2.02, 1.25	0.647	− 1.18	− 2.51, 0.14	0.080
Current perceived health status	Very good/good	Ref			Ref		
	Fair/poor	− 5.14	− 6.89, − 3.38	< 0.001	− 5.44	− 6.82, − 4.06	< 0.001
Duration (months) since discharged from ETC		− 0.07	− 0.29, 0.14	0.502	− 0.05	− 0.23, 0.12	0.536
Time spent at ETC		− 0.08	− 0.15, − 0.01	0.018	− 0.09	− 0.15, − 0.04	< 0.001
Number of post-Ebola symptoms		− 1.45	− 1.88, − 1.01	< 0.001	− 0.96	− 1.32, − 0.60	< 0.001
Arthralgia (Joint pain)	Yes	− 2.69	− 5.29, − 0.10	0.042	− 1.16	− 3.28, 0.96	0.283
	No	ref			ref		
Headache	Yes	0.08	− 1.82, 1.99	0.931	− 0.73	− 2.27, 0.82	0.357
	No	Ref			Ref		
Ocular symptoms	Yes	− 3.77	− 5.36, − 2.17	< 0.001	− 2.55	− 3.86, 1.24	< 0.001
	No	Ref			Ref		
Fatigue	Yes	− 2.62	− 4.22, − 1.01	0.001	1.08	− 2.39, 0.24	0.109
	No	Ref			Ref		
Back pain	Yes	− 1.51	− 3.13, 0.11	0.067	− 0.85	− 2.17, 0.47	0.205
	No	Ref			Ref		
Abdominal pain	Yes	− 0.52	− 2.20, 1.17	0.544	− 1.08	− 2.44, 0.29	0.121
	No	Ref			Ref		
Auditory symptoms	Yes	− 2.19	− 4.34, − 0.04	0.046	− 1.28	− 3.03, 0.48	0.153
	No	Ref			Ref		
Skin disorders	Yes	− 3.64	− 5.86, − 2.140	0.001	− 2.16	− 3.98, 0.34	0.020
	No	Ref			Ref		
Alopecia	Yes	− 1.37	− 4.01, 1.26	0.306	− 0.85	− 2.99, 1.30	0.438
	No	Ref			Ref		
Internalized stigma (negative self-perception subscale		− 2.89	− 3.90, − 1.87	< 0.001	− 2.68	− 3.50, − 1.86	< 0.001
Enacted stigma		− 3.17	− 4.46, − 1.88	< 0.001	− 2.58	− 3.63, − 1.53	< 0.001

**Table 4:** Multiple linear regression analysis to identify factors associated with Physical Health (PCS) among Ebola survivors (N = 358)

Characteristics	Variable	Unstandardized coefficients		95%CI		*p* value
		β	SE	Lower	Upper	
Sex	Female	Ref		–		
	Male	− 0.04	0.76	− 1.54	1.45	0.956
Age group	18–49	Ref				
	≥ 50 years	− 3.90	1.31	− 6.47	− 1.32	0.003
Educational status	Non-formal	− 2.80	1.202	− 5.160	− 0.43	0.021
	Primary	− 0.25	1.44	− 3.09	2.59	0.862
	Secondary	− 0.79	1.232	− 3.21	1.635	0.523
	Tertiary	Ref				
Marital status	Single	Ref				
	Married/cohabitating	0.48	0.946	− 1.38	2.34	0.614
	Divorce/separated/	− 1.51	3.407	− 8.21	5.20	0.659
	widowed	0.47	1.19	− 1.86	2.81	0.691
Region	Northern region	− 5.47	0.90	− 7.25	− 3.69	< 0.001
	Southern region	− 1.66	1.13	− 3.88	0.57	0.144
	Eastern region	0.09	1.19	− 2.24	2.43	0.937
	Western area	Ref				
Perceived health status	Fair/poor	− 3.68	0.87	− 5.38	− 1.98	< 0.001
	Very good/good	Ref				
Number of post Ebola symptoms reported		− 1.08	0.33	− 1.74	− 0.431	< 0.001
Time spent at ETC		− 0.01	0.03	− 0.06	0.05	0.805
Ocular symptoms	Yes	− 1.077	0.804	− 2.659	0.51	0.181
	No	Ref				
Fatigue	Yes	− 1.71	0.74	− 3.17	− 0.25	0.022
	No	Ref				
Skin disorder	Yes	− 0.45	1.08	− 2.58	1.68	0.676
	No	Ref				
Arthralgia (joint pain)	Yes	1.05	1.22	− 1.36	3.45	0.392
	No	Ref				
Auditory symptoms	Yes	− 0.08	1.04	− 2.13	1.96	0.936
	No	Ref				
Internalised stigma (negative self-perception)		− 0.10	0.61	− 1.29	1.09	0.875
Enacted stigma		− 2.61	0.72	− 4.02	− 1.20	< 0.001

Table 5 shows the demographic, health-related, and psycho-social factors associated with the mental health composite score among Ebola survivors. EVD survivors with a unit increase in the time spent in the Ebola treatment centre were likely to report a decrease in mental health (β = − 0.60, 95% CI − 0.103 to− 0.18, *p* = 0.006). Also, EVD survivors who experienced a unit increase in Enacted Stigma were more likely to report a decreased level of mental health (β = − 1.50, 95% CI − 2.67to − 0.33, *p* = 0.012).

**Table 5:** Multiple linear regression analysis to identify factors associated with MCS among Ebola survivors (N = 358)

Characteristics	Variable	Unstandardized coefficients		95%CI		*p* value
		β	SE	Lower	Upper	
Age group	≥ 50 years	− 2.61	1.10	− 4.77	− 0.44	0.018
	18–49 years	Ref				
Educational status	Non-formal	− 1.76	0.10	− 3.73	0.20	0.078
	Primary	− 0.21	1.20	− 2.57	2.16	0.863
	Secondary	− 1.19	1.03	− 3.22	0.847	0.252
	Tertiary	Ref				
Marital status	Single	Ref				
	Married/cohabitating	0.53	0.79	− 1.02	2.08	0.500
	Divorce/separated/	3.30	2.85	− 2.30	8.90	0.247
	widowed	0.58	0.99	− 1.36	2.52	0.556
Region	Northern region	− 3.03	0.76	− 4.53	− 1.54	< 0.001
	Southern region	− 2.09	0.94	− 3.95	− 0.24	0.027
	Eastern region	0.54	0.96	− 1.34	2.43	0.571
	Western area	Ref				
Employment status after surviving Ebola	Not employed	− 0.65	0.65	− 1.92	0.62	0.318
	Employed	Ref				
Perceived health status	Fair/poor	− 4.28	0.72	− 5.69	− 2.88	< 0.001
	Very good/good	Ref				
Time spent at ETC (days)		− 0.04	0.02	− 0.09	0.01	0.098
Number of post ebola symptoms reported		− 0.60	0.22	− 1.03	− 0.18	0.006
Ocular symptoms	Yes	− 0.76	0.65	− 2.03	0.51	0.239
	No	Ref				
Skin disorder	Yes	− 0.20	0.90	− 1.97	1.57	0.822
	No	Ref				
Enacted stigma		− 1.50	0.59	− 2.67	− 0.33	0.012
Internalised stigma (negative perception)		− 0.52	0.50	− 1.51	0.47	0.300

## Discussion

Our study sought to explore the association between social factors (demographic, health-related, and psycho-social) and HRQoL among Ebola survivors. We found a low physical health and mental health summary scores, indicating that EVD survivors maybe suffering from poor physical and mental health. This finding is in line with previous Sierra Leonean studies, which show that EVD survivors continue to experience poor physical and mental health more than two and half years after discharge from the Ebola treatment centre [11, 12, 38]. EVD survivors continue to suffer from psychological distress due to grief resulting from losing loved ones, social exclusion, and community stigmatisation [6, 23]. Furthermore, we observed a lower score for physical role limitation, a key component of the physical health in our study, suggesting that EVD survivors may face limitations in performing basic daily physical activities. Mobility limitation has been reported to be a common post-Ebola disability. For example, a previous study in Sierra Leone has found that EVD survivors had significant limitations in walking and climbing stairs a year after recovery, and musculoskeletal pain was a contributing factor [9]. Regarding the mental health of EVD survivors, recent Sierra Leonean studies have shown that anxiety, depression, post-traumatic stress disorder and stigma are common among EVD survivors [10, 11, 38]. Our findings regarding the physical and mental health limitations among EVD survivors further emphasised the need for them to access comprehensive healthcare that includes specialist care and mental health services [39, 40].

In our study, age, educational status, the burden of post-Ebola symptoms, and enacted stigma were significantly associated with a decreased physical component score depicting poor physical health of EVD survivors. Our study shows that older EVD survivors had higher odds of having decreased levels of mental health. Similar findings have been reported among patients living with chronic health conditions, in which older patients were more likely to report poorer quality of life than younger ones [41, 42]. However, our result was inconsistent with a recent Chinese study among COVID-19 survivors, which reported no association between age and physical component score [19] which may reflect differences in biologic and socio-cultural contexts. The link between age and physical health in our study may be due to the physiological changes accompanying old age and the loss of capacity to fully undertake activities of daily living as well as the incremental activities of daily tasks. Also, older individuals are more likely to suffer from chronic pain, experience challenges in pain management, leading to functional impairment, falls, depression, and sleep disturbance [43, 44]. Regarding gender, a recent study among COVID-19 survivors reported that being female was a significant determinant of reduced physical health [19]. This prior observation is inconsistent with our finding in which there was no significant gender difference. We also observed in our study that EVD survivors with no formal education were more likely to report decreased physical health compared to those with college education. A similar finding was reported among patients with stroke at tertiary level hospital in Ethiopia in which stroke survivors who did not go to school were likely to report lower physical health scores than those that went to school [45]. Our finding might be explained in that EVD survivors who cannot read and write have lower levels of understanding of their physical health and psychological issues and inability to make informed decisions regarding the choice of appropriate treatment options, including selfcare.

We also observed that as the burden of post-Ebola symptoms increases, physical and mental health of EVD survivors decreases, suggesting that the higher the number of post-Ebola physical health symptoms a survivor has, the more significant the impact on his/her HRQoL. A similar finding has been reported among COVID-19 survivors [20] and co-morbid diabetic patients [41]. Also, previous research has reported a synergic relationship between physical symptom burden and mental health [46]. Our finding suggests the need for support for clinicians to consider the potential impact of post-Ebola physical symptom burden on EVD survivors’ mental wellbeing.

Previous research has reported that individuals with more chronic physical health symptoms are more likely to have limited physical activities leading to less social interaction, and these factors are linked to low quality of life [47]. Consistent with finding from a HRQoL study among patients with chronic disease [41], EVD survivors in our study were more likely to report decreased physical and mental health as the level of stigmatisation from the public increases. Our finding suggests that family and community stigmatisation, ostracisation and discrimination negatively affected EVD survivors' physical and mental health. The association between enacted stigma and mental health in our study suggests that EVD survivors may start to experience feelings of shame, guilt, worthlessness, poor self-esteem and suicidal thoughts due to being are ostracised and rejected by the public, leading to poor mental health. Similar findings have been reported among HIV/AIDS patients, in which internalised stigma was related to lower emotional wellbeing [48]. Our finding may be explained in that stigma has been reported to be common among survivors of emerging and re-emerging infectious disease and, it has been reported to be associated with greater psychiatric symptoms, including depression and post-traumatic stress disorder [10, 49]. Community stigmatisation, discrimination and ostracisation affect patient's help- and treatment-seeking behaviours and treatment adherence, thereby putting their health at risk [10, 50–52] and, this might explain our result. Anti-stigma interventions in the form of mental health literacy campaigns (implemented by government or non-governmental organisations) can provide correct information about the stigmatised condition aimed at correcting misinformation, dispelling myths, and/or contradicting negative attitudes and beliefs [53]. Also, peer support and interventions that will allow contact between EVD survivors and the public to overcome the existing interpersonal divide and foster positive connection and interaction [53] can be considered by government or non-governmental agencies to help reduce stigma towards EVD survivors and invariably improve their overall wellbeing. Given the link between public stigma and decreased physical and mental health in our study, anti-stigma and discriminatory laws need to be enacted by the government and local authorities at the national and community levels to prevent and protect EVD survivors against community stigmatisation and ostracisation, which will in turn help improve their HRQoL.

Findings from our univariate analysis indicate that the longer the time spent by an EVD survivor as a patient at an Ebola treatment centre was associated with decreased in mental health although there was no statistically significant association in the multivariate analysis. Our finding is in line with a recent study among COVID-19 survivors, in which no significant association existed between mental health component score and length of stay at the COVID-19 treatment centre [19]. Our finding might be due to the traumatic events EVD survivors experience while being admitted at an Ebola treatment centre. These traumatic events include flashbacks associated with witnessing fellow EVD patients dying, the uncertainty around mortality associated with living with EVD and, feelings of loneliness and isolation [6]. A previous study has reported high prevalence mental health related symptoms among EVD patients during their time at ETC [54]. Further research is required to explore the impact length of stay at an Ebola treatment centre has on mental health among EVD survivors and survivors of other emerging infectious disease.

Our study has certain limitations that readers should consider when interpreting the findings. A cause-effect relationship could not be inferred, given that we employed a cross-sectional design in our study. A longitudinal study is required going forward to determine whether HRQoL improves with time given that our study employed a cross-sectional design. A future study should compare HRQoL scores with the general population since our study did not compare scores to the HRQoL of the general Sierra Leonean population. There is a tendency for recall bias, given that the data were based on retrospective self-report. Our study failed to assess EVD survivors’ level of social support, which can be a potential cofounder of HRQoL. Also, the non-linear relationship between factors such as age, symptom burden and HRQoL should be further explored in future studies. Notwithstanding these limitations, our findings have relevance to EVD survivors in Sierra Leone since a nationwide sample was used in our study.

## Conclusions

Our study has improved our understanding of how socio-demographic, health related, and psycho-social (Ebola-related stigma) factors might influence the HRQoL of EVD survivors in Sierra Leone. Role limitation-physical, and role limitation-emotional were the most affected domains suggesting that EVD survivors maybe suffering from poor physical and mental health. Age, post-Ebola symptom burden and Ebola-related stigma were associated with decreased levels of physical and mental health of EVD survivors, and these characteristics should be considered by healthcare professionals, including mental health providers as possible risk factors for EVD survivors' HRQoL. These findings emphasised the need for EVD survivors to access comprehensive healthcare that includes specialist care and mental health services. Also, community-driven stigma reduction strategies such as psychoeducation, cognitive techniques, peer support, legislative and policy change at the local and national level all need to be explored to improve EVD survivors’ HRQoL.

## Data Availability

Due to confidentiality and privacy concerns, and given the sensitivity surrounding stigma and discrimination among Ebola survivors, our study did not receive approval from the Sierra Leone Ethics and Scientific Review Committee to publicly share the raw data. Also, Ebola survivors consented to participate in the study on the basis that their data would not be shared with anyone except members of the research team. However, upon reasonable request, the anonymised raw data underlying the findings of this study can be made available through the corresponding Author.

## Abbreviations

EVD: Ebola virus disease
ETC: Ebola treatment centre
HRQoL: Health related quality of life
